# Unraveling the evolutionary dynamics of toxin-antitoxin systems in diverse genetic lineages of *Escherichia coli* including the high-risk clonal complexes

**DOI:** 10.1128/mbio.03023-23

**Published:** 2023-12-20

**Authors:** Anuradha Singh, Aditya Kumar Lankapalli, Suresh Kumar Mendem, Torsten Semmler, Niyaz Ahmed

**Affiliations:** 1 Pathogen Biology Laboratory, Department of Biotechnology and Bioinformatics, University of Hyderabad, Hyderabad, Telangana State, India; 2 Department of Biology and the Ineos Oxford Institute for Antimicrobial Research, University of Oxford, Oxford, United Kingdom; 3 Robert Koch Institute, Berlin, Germany; Fondazione Biotecnopolo di Siena, Siena, Italy

**Keywords:** *Escherichia coli*, toxin-antitoxin (TA) systems, sequence type (ST), antimicrobial resistance, virulence, adaptation

## Abstract

*Escherichia coli* is a highly versatile microorganism with a unique ability to survive and persist in varied niches of the human and animal hosts and the environment. While commensal strains of *E. coli* play a crucial role in preserving and maintaining the balance and function of their community within the gut microflora, pathogenic strains are often implicated in a wide range of infections and outbreaks, posing a serious threat to public health systems. With the increasing burden of highly virulent and antimicrobial-resistant *E. coli* infections, it is imperative to understand the host-defense mechanisms of bacteria from all possible dimensions. Mobile genetic elements (MGE)-mediated acquisition of genetic material, chromosomal reduction, and genome optimization are three important events that play a significant role in bacterial evolution and enable them to survive in diverse environmental niches. Toxin-antitoxin (TA) systems are genetic elements that help in the maintenance of MGEs and are often associated with stress response phenotypes, including antimicrobial resistance. In this study, large-scale comparative genomics of 950 genomes spanning 19 different sequence types (STs) (eight phylogroups) revealed ST-wide prevalence patterns of TA systems with a median of 23 toxin groups per strain. Our analyses revealed significant genomic reduction in the members of phylogroup B2 (ST131, ST95, ST73, ST12, and ST127) and phylogroup C (ST410) as evident from a diminished toxin repertoire amidst abundant orphan antitoxins. Moreover, our observations also enabled crucial insights into the copy number of toxin groups, the genetic organization of TA operons, and their association with other genetic coordinates (antimicrobial resistance encoding genes/virulence genes/mobile genetic elements). By unraveling the association of the genetic coordinates/STs with the toxin groups, this study significantly boosts our understanding of the functional implications of TA systems in different evolutionary contexts entailing pathogenic *Escherichia*.

**IMPORTANCE:**

Large-scale genomic studies of *E. coli* provide an invaluable opportunity to understand how genomic fine-tuning contributes to the transition of bacterial lifestyle from being commensals to mutualists or pathogens. Within this context, through machine learning-based studies, it appears that TA systems play an important role in the classification of high-risk clonal lineages and could be attributed to their epidemiological success. Due to these profound indications and assumptions, we attempted to provide unique insights into the ordered world of TA systems at the population level by investigating the diversity and evolutionary patterns of TA genes across 19 different STs of *E. coli*. Further in-depth analysis of ST-specific TA structures and associated genetic coordinates holds the potential to elucidate the functional implications of TA systems in bacterial cell survival and persistence, by and large.

## INTRODUCTION

*E. coli* strains form the most widely studied members of *Enterobacteriaceae* that naturally associate with the gastrointestinal tracts of humans and animals. Although the commensal strains of *E. coli* preserve and maintain the balance and functional acumen of their niches within the gut microbial communities, their pathogenic forms lead to a wide range of intestinal and extraintestinal infections (1). Some common diseases caused by *E. coli* include urinary tract infections (UTIs), bloodstream infections (BSI), and meningitis (1). In recent times, *E. coli* has garnered significant attention in the global public health domain as a major pathogen emerging and disseminating in the form of multidrug-resistant (MDR) lineages such as ST131 and ST410 (2, 3). According to informed estimates, if the situation remains uncontrolled, antimicrobial resistance (AMR) can lead to 10 million deaths by 2050 surpassing all other fatalities (4, 5). In response, there is an urgent need to understand the intricate molecular mechanisms underlying the emergence and dissemination of the MDR pathogens and their lineages. Since the bacterial TA systems are anticipated and shown to play significant roles in the emergence, maintenance and dissemination of AMR and other virulence traits (6–14), research on MDR/AMR phenotypes in bacteria is expected to focus on the underlying mechanism through exploring TA systems at population levels.

TA systems are bicistronic operons found extensively in bacterial genomes. A TA operon usually consists of two components: a stable toxin encoded by a downstream gene and its cognate unstable antitoxin encoded by an upstream gene (6). Under normal bacterial growth conditions, antitoxin binds to its cognate toxin to neutralize it and promote bacterial growth. Whereas, under stress conditions, stress-induced bacterial proteases degrade antitoxin and release functional toxin to inhibit bacterial growth. Under these conditions, a fraction of bacterial population (around 1%) becomes sub-dormant and persister cells escape the effect of antimicrobials without any changes discerned in their genes. Thus, TA systems play an important role in cell survival and AMR under environmental stress by inducing a persister state (7). Originally, TA systems were believed to play an important role in plasmid maintenance via the “post-segregational killing (PSK)” process (8). However, after four decades of research on TA systems, mounting evidence suggests their role in the maintenance of other genetic elements, such as genomic islands, integrative conjugative elements, and prophages (9). For instance, the *Salmonella* genomic island 1 (SGI1)-encoded TA system (SgiA/SgiT) plays an important role in the maintenance of this MDR-encoding island inside the host cell chromosome, particularly when IncA/C is also present (10). Furthermore, TA systems have also been linked to bacterial virulence, AMR, and phage suppression (7, 11). Based on the nature of the antitoxin (RNA/protein) and its interaction with the toxin component, TA systems have been classified into eight different categories (12). Out of the different types of TA systems, type II TA systems are highly prevalent and diverse and found in bacterial as well as archaeal members (12). So far, researchers have identified 36 potential TA systems in the *E. coli* K-12 genome, out of which eight have been well characterized; these include MazE/MazF, RelE/RelB, ChpBK/ChpBI, YafQ/DinJ, YoeB/YefM, HipA/HipB, YafO/YafN, and MqsR/MqsA (13). Among the *Mycobacterium* species, it has been observed that *Mycobacterium tuberculosis* encodes over 60 TA systems, whereas the nonpathogenic *M. smegmatis* harbors only two TA systems (13). This variation in the prevalence of TA systems across pathogenic and nonpathogenic members suggests a possible association of the TA systems with bacterial virulence (14).

Over the years, high-throughput genomics has remained pivotal in understanding the prevalence and genetic diversity of bacterial species such as *E. coli* (2, 15). Our previous study on *E. coli* revealed significant diversity in terms of their genomic features (AMR genes, virulence genes, mutations, integrons, transposons, and plasmids) across 19 STs (16). Furthermore, the study provided initial working evidence towards the clonal evolution of *E. coli*. Machine learning-based predictive modeling identified 86 key ST-specific signatures, including TA systems, that could be implicated in context-specific adaptation strategies, including drug tolerance (16).

Building on the above findings, the current study presents a comprehensive, in-depth analysis of TA systems by harnessing the power of a strong collection of 5,653 *E. coli* genomes from where an eventual total of 950 high-quality genome sequences were recruited after rigorous quality controls based on machine learning-guided classifications, as also described in our earlier study (16). While a few experimental and bioinformatics-based studies paved the way for exploring TA systems in important bacterial pathogens such as *E. coli*, *Klebsiella pneumoniae*, and *Pseudomonas syringae* (17–19), the other works used specific experimental or applied approaches in the context of a particular *E. coli* pathotype (20, 21). The present study aimed to provide a comprehensive and global perspective on TA systems in *E. coli* genomes by utilizing high-throughput genomic data representing 19 different STs. We attempted to investigate the diversity of TA systems, including those of type II and type IV, on a population-wide scale and unraveled their distribution and evolutionary patterns among different STs/phylogroups. Our analyses revealed that members of phylogroups B2 and C exhibit reduced TA repertoires thereby strengthening the idea that gene loss confers fitness advantages to certain specialist lineages. Overall, our study sheds light on preponderances of TA systems in the studied bacteria and the genetic processes that drive their evolution, with potential implications relevant to the adaptation strategies of a major human-animal pathogen such as *E. coli*.

## RESULTS

### Curation and characterization of high-quality genomes to predict toxin-antitoxin pairs

**Fig 1:**
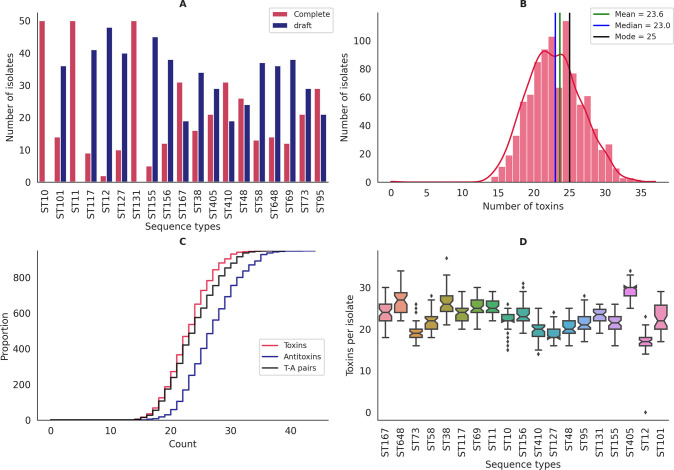
(**A**) Grouped bar plot depicting the genome status of isolates under study. (**B**) Histogram depicting the status of toxins/isolate. (**C**) ECDF plot depicting isolates falling below each unique value (number of toxins/antitoxins/TA pairs) in the data set. (**D**) Boxplot depicting the number of toxins per strain stratified by *E. coli* STs.

Bacteria, mostly of the pathogenic *E. coli* clan, were studied for TA pairs based on a total of 950 genome assemblies consisting of 416 complete and 534 draft genomes (File S1). This was done with the help of a curated and quality-controlled set of high-quality genomes categorized based on STs by machine learning algorithms (16) trained on thousands of genomes from multiple hosts as well as different regions of the world, thus providing a global subsample for our analyses (File S1) spanning 19 important STs across eight phylogroups. Among these, ST10 (phylogroup A), ST11 (phylogroup E), and ST131 (phylogroup B2) were represented solely by complete genomes, whereas good-quality draft genomes were used, when required, for the rest of the STs to reach a total count of 50 genomes per ST (Fig. 1A). The size of the draft genomes ranged from 5.1 MB to 5.9 MB, with the number of contigs/scaffolds restricted to a total of 192 or fewer. These draft genomes showed an average G+C content of 50.59%, and the minimum N50 value was observed to be 53,400 bp.

SLING predicted 169 toxin groups (hits or “H”) and 290 antitoxin groups (partners or “P”) in 950 isolates studied, with a wide range of diversity among different STs/phylogroups (File S2 provides details regarding various key terms including H and P). As a result of different hits and partner combinations, a total of 314 TA pairs were predicted. In a complete data set of 950 isolates, toxins were detected with a median value of 23 (range 0–37), which means typically an isolate harbors 23 toxins (Fig. 1B). The number of toxins per strain considerably varied from a minimum of zero in a strain from ST12 to as many as 37 in a strain from ST38 (Fig. 1D; File S3). Notably, all the STs under the study were replete with toxins with a minimum of 14 toxins per strain except one of the isolates belonging to ST12 (in-house ID, ST000120035). Compared with other STs, ST405 and ST38 from phylogroup D and ST648 from phylogroup F exhibited a higher average number of toxins per strain (Fig. 1D; File S3).

### ST and phylogroup specific prevalence patterns of toxins

**Fig 2:**
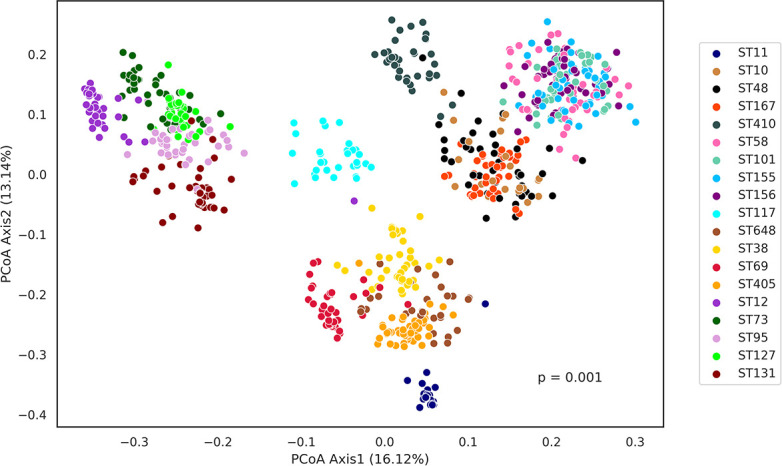
PCoA based on the Jaccard distance matrix of toxin repertoire colored by STs. Isolates belonging to a particular ST tend to cluster together. Moreover, members of the same phylogroup also cluster closely, depicting their evolutionary relatedness.

Our principal coordinate analysis (PCoA) results based on the Jaccard distance matrix of toxin repertoires revealed that isolates belonging to a particular ST tend to cluster together (Fig. 2). The top two ordination axes from the PCoA explained a significant proportion of the variance (29.26%) observed in the data (Fig. S1). Furthermore, it was also observed that STs belonging to the same phylogroup also cluster near each other and, in some cases, they overlap, suggesting genetic similarity at the phylogroup level (Fig. 2). Permutational analysis of variance (PERMANOVA) revealed a *P* value of 0.001, indicating that STs significantly differed from each other based on their toxin repertoire.

In terms of toxin groups, the ParE domain was observed to have a maximum number of groups, i.e., 22, followed by GP49 and HicA (File S3). On the other hand, DUF4258, MqsR, CptA_toxin, Toxin_yhaV, HipA N-terminal domain, HipA_C-Couple_hipA, GnsAB_toxin, yjhX_toxin, and YoeB_toxin had only single group.

**Fig 3:**
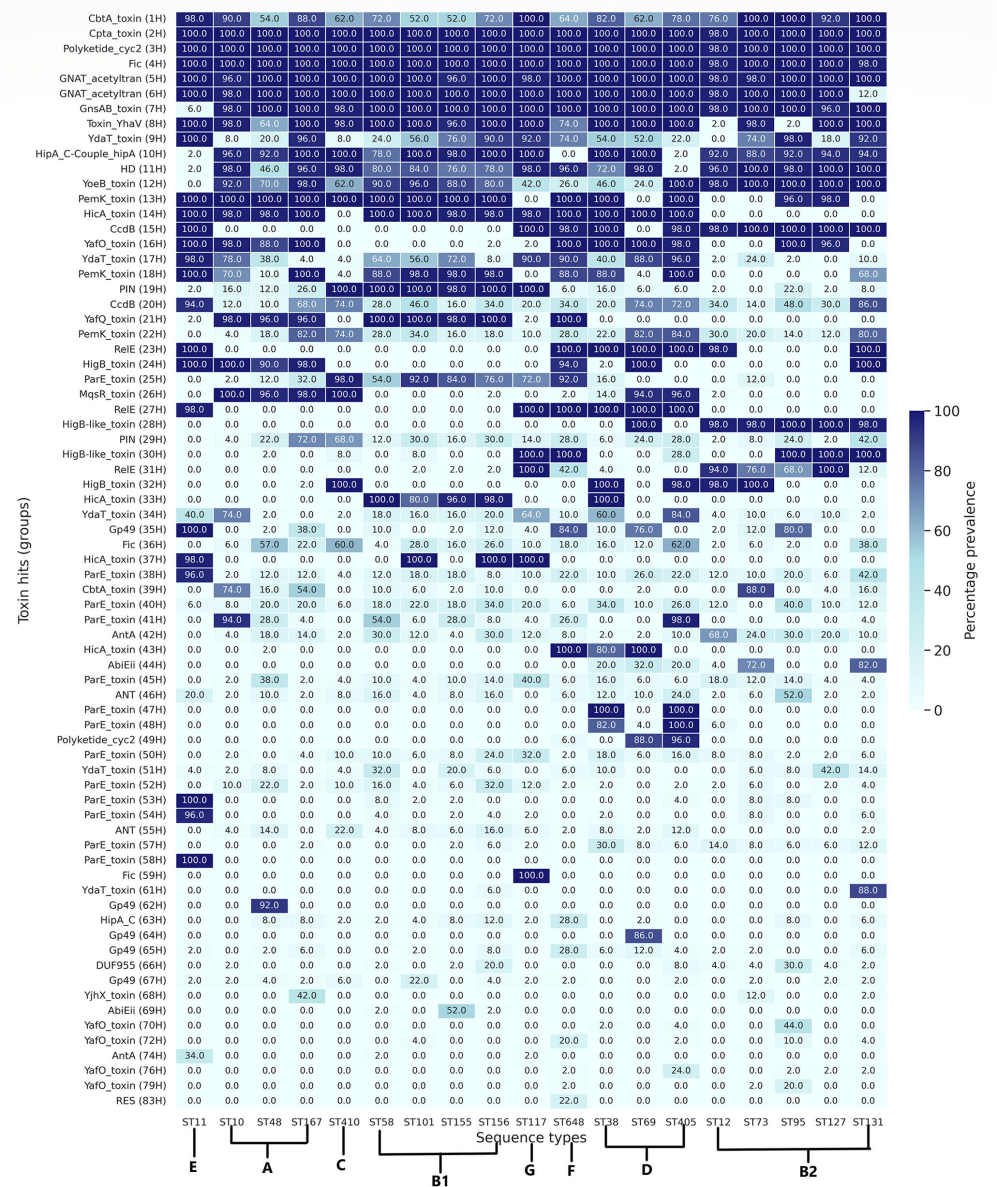
Heatmap illustrating the percentage prevalence of toxin groups across 19 STs of *E. coli*. The *x*-axis depicts ST lineage, while the *y*-axis depicts toxin groups identified. The color bar on the right side depicts % prevalence.

Across all the STs studied, a ST-specific and phylogroup-specific prevalence pattern of toxins was observed (Fig. 3; File S3). Four toxin hits [CptA_toxin (2H), Polyketide_cyc2 (3H), Fic (4H), and GNAT_acetyltransferase (5H)] out of 169 were highly prevalent (≥80%) across all the STs under the study. Out of these four, domains of three groups [Polyketide_cyc2 (3H), Fic (4H), and GNAT_acetyltransferase (5H)] were associated with type II TA systems, while CptA _toxin (2H) belonged to type IV TA systems (22–24). Several groups were found to be highly prevalent in all STs except a few. For instance, GNAT-acetyltransferase (6H), known to inhibit translation via acetylation of aminoacyl-tRNAs, was highly prevalent in all the isolates but only rarely present in those belonging to ST131 (12%) (24). Similarly, GnsAB_toxin (7H) was rare in ST11 (6.0% of isolates) but prevalent (>80% of the isolates) across the rest of the STs. Toxin-YhaV (8H), responsible for ribosome-dependent cleavage of mRNA, was prevalent (≥60%) in all the STs under study except ST95 (2.0%) and ST12 (2.0%) of phylogroup B2 (25). HipA_C-Couple_hipA (10H) was highly prevalent in all the STs except ST648, ST405, and ST11, where it was found in less than or equal to 2% of the isolates. YoeB_toxin (12H), which inhibits translation initiation, was observed to be exclusively absent from ST11 (26). Hits corresponding to the HicA domain (14H, 33H, 37H, and 43H) were exclusively rare (≤20%) from the members of phylogroup B2 (ST12, ST73, ST95, ST127, and ST131) and phylogroup C (ST410). CcdB (15H) was completely absent from the STs belonging to phylogroups A, C, and B1 and ST69 of phylogroup D. YafO (16H) was observed with lower prevalence in the members of phylogroups C, B1, and G and ST131 and ST73 of phylogroup B2. On the other hand, PIN (19H) was found to be highly prevalent across STs belonging to phylogroups C, B1, and G, while being rare in other STs. YafQ (21H) was observed to be highly prevalent across STs belonging to phylogroups A, B1, and F. Hits corresponding to the RelE domain were exclusively absent from the STs belonging to A, C, and B1. HicA (33H) toxin was highly prevalent across the members of phylogroups B1 and ST38 of phylogroup D. AbiEii (44H) was observed to be present in 72% of the strains of the ST73 and 82% of strains of the ST131. In addition to the shared patterns, unique ST-specific patterns were also observed. One of the interesting observations was that Fic (59H) was exclusively found in ST117 and absent in all other STs. Similarly, Gp49 (62H) was exclusively observed among the isolates of ST48.

### Copy number variation in toxin hits

**Fig 4:**
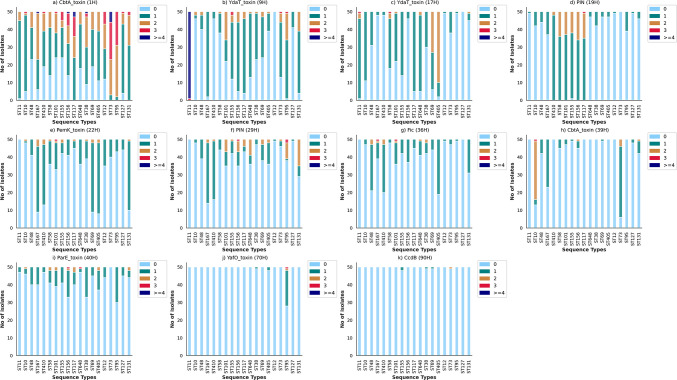
Stacked bar plots depicting copy number variation of 11 toxin groups in 19 STs studied. (a) CbtA_toxin (1H), (b) YdaT_toxin (9H), (c) YdaT_toxin (17H), (d) PIN (19H), (e) PemK_toxin (22H), (f) PIN (29H), (g) Fic (36H), (h) CbtA_toxin (39H), (i) ParE_toxin (40H), (j) YafO_toxin (70H), and (k) CcdB (90H).

In-depth analysis of toxin hits provided valuable insights into the copy number of hits found per strain. Many different hits were found to be present in multiple copies. The top 11 toxin hits, namely, CbtA_toxin (1H), YdaT_toxin (9H), YdaT_toxin (17H), PIN (19H), PemK_toxin (22H), PIN (29H), Fic (36H), CbtA_toxin (39H), ParE_toxin (40H), YafO_toxin (70H), and CcdB (90H), showing variation in copy number are presented in Fig. 4. The copy number of CbtA_toxin (1H), a dual inhibitor of cell division and elongation, was discovered to vary greatly across various STs (27). Compared with other STs, ST73 showed a maximum copy number of CbtA_toxin (1H), i.e., 8 (File S4). Also, all the isolates belonging to ST73, ST95, ST131, and ST117 were positive for this particular hit. However, in other STs, isolates showed a complete absence of CbtA_toxin (1H). Another important finding concerning the copy number was associated with YdaT_toxin (9H); isolates belonging to ST11 had at least three copies of this particular hit, and 49/50 isolates had a copy number ≥ 4. STs from phylogroups B1, C, and G had a greater copy number of PIN (19H) than the other phylogroups.

### Genetic organization of toxin-antitoxin pairs

**Fig 5:**
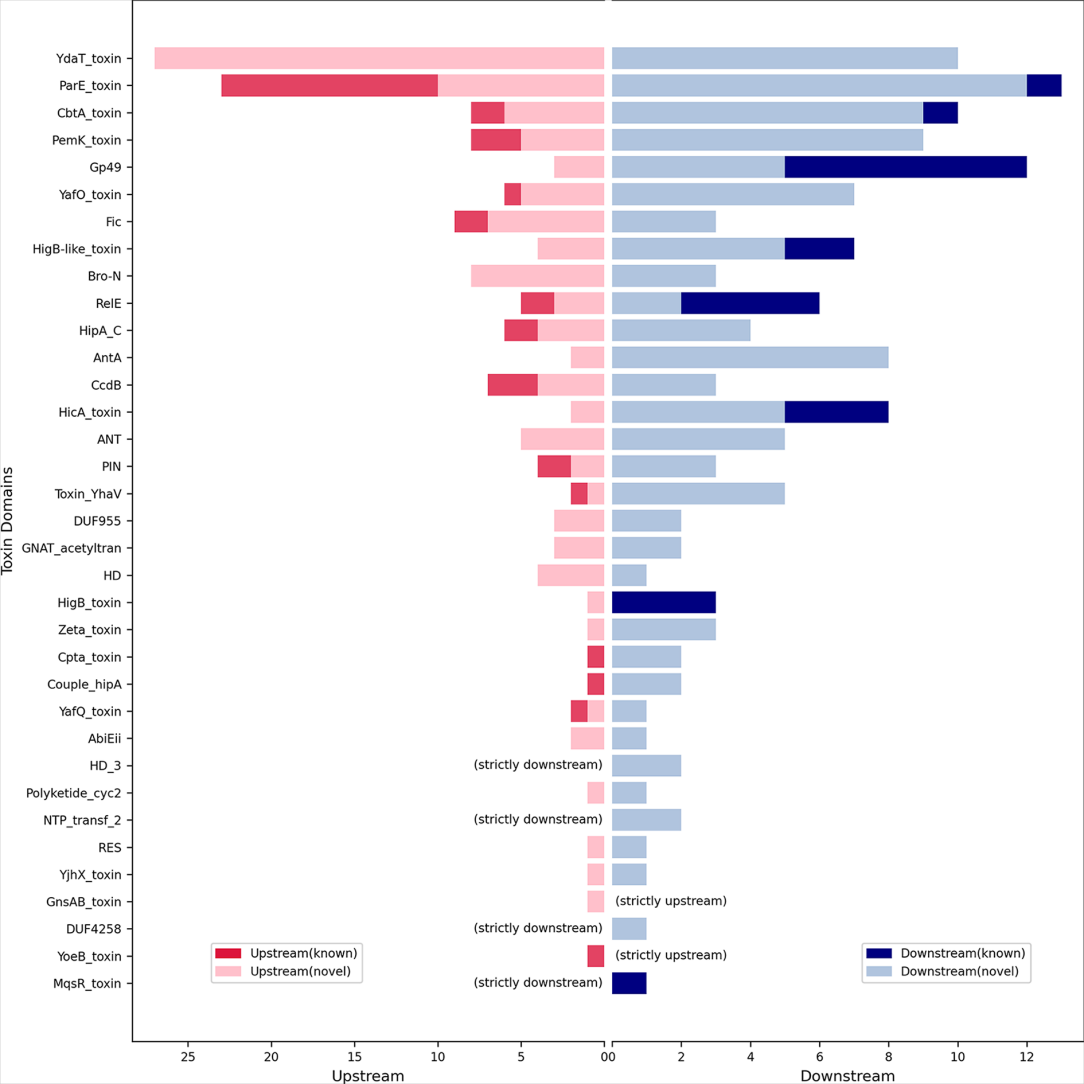
Double-sided bar plot depicting the genetic orientation of TA operons. Here, the *x*-axis depicts the orientation of antitoxins (upstream/downstream) along with their count, while the *y*-axis depicts Pfam profiles of toxins. Red-colored bars on the left side depict known antitoxins positioned upstream, while light-red bars indicate novel antitoxins positioned upstream. Similarly, blue-colored bars on the right side depict known antitoxins positioned downstream, while light-blue bars indicate novel antitoxins positioned downstream.

In most cases, particularly in type II TA systems, the antitoxin genes are found upstream of the toxin gene (28). In our data set, in addition to the canonical AT-T (antitoxin-toxin) organization, two other kinds of genetic organizations, i.e., T-AT (toxin-antitoxin) and AT-T-AT (antitoxin-toxin-antitoxin), were also observed. The reverse organization (T-AT) of TA operons has been reported previously in various members of *Enterobacteriaceae*, such as *E. coli* and *K. pneumoniae* (18, 29). It was observed that a single toxin could be associated with different types of antitoxins in different orientations. We also found partners (antitoxins) associated with domains DUF4258, NTP_transf_2, HD_3, and MqsR_toxin positioned strictly downstream (Fig. 5). In comparison, two domains, YoeB_toxin and GnsAB_toxin, had their partners strictly upstream, while the rest (29) of the domains had antitoxins in both orientations (Fig. 5). Despite the fact that our data set contained antitoxin positions in both the upstream and downstream directions, the upstream antitoxin position was found to be more prominent.

### Prevalence patterns of partners (antitoxins) and complete TA operons

**Fig 6:**
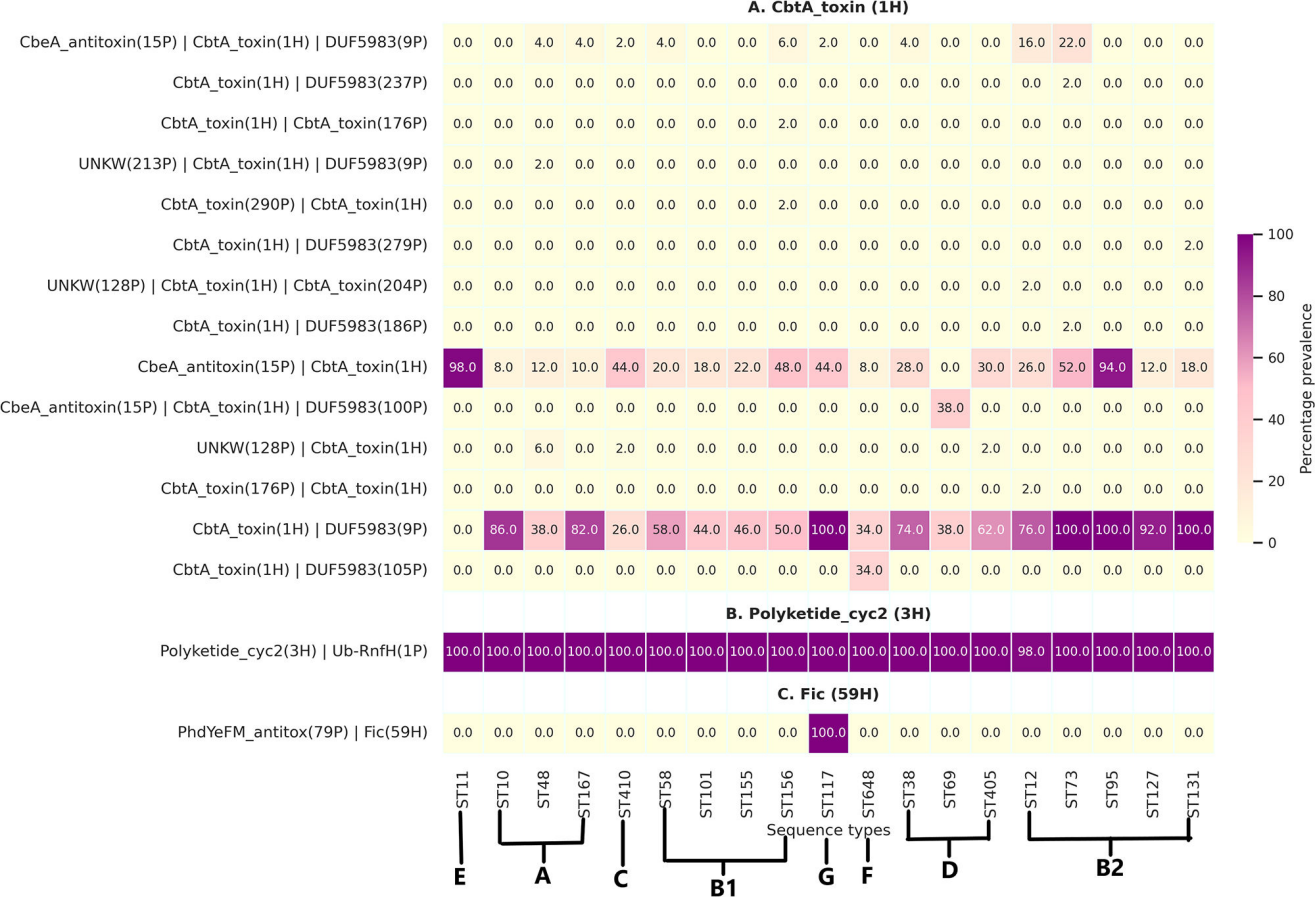
Heat maps depicting the genetic organization of TA structures and mosaic nature. (**A**) Cbta_toxin (1H), (**B**) Polyketide_cyc2 (3H), and (**C**) Fic (59H).

SLING-based prediction of linked genes in our data set revealed higher diversity of antitoxin partners in TA operons as compared with toxin groups (Fig. S2 and S3). The majority of antitoxins appear to overlap within the 50-bp upper or lower distance from a toxin. It was observed that a single toxin group could be associated with varying numbers of partners, ranging from a minimum of one partner to a maximum of 13 partners in the genomic data set (File S5; Fig. S4). For instance, YdaT (9H) toxin was observed to be associated with 13 different partners in two different genetic orientations, i.e., AT-T and AT-T-AT. Similarly, Fig. 6A represents the different genetic orientations of the partners associated with the CbtA_toxin (1H). In the case of partners associated with CbtA_toxin (1H), it was observed that the TA structure represented by CbtA_toxin (1H)/DUF5983 (9P) was observed across all the STs except ST11. However, isolates belonging to ST11 showed a positive association with the CbeA_antitoxin (15P)/CbtA_toxin (1H). Furthermore, whenever CbeA_antitoxin (15P) was linked with CbtA_toxin (1H), it was consistently positioned upstream to the toxin hit. Conversely, when DUF5983 (9P) was associated with CbtA_toxin (1H), it was always located downstream of the toxin hit. Hits such as Polyketide_cyc2 (3H) (Fig. 6B) were consistently associated with a single type of partner and exhibited high prevalence across different STs. Overall, the association between these hits and their antitoxin partners is more dynamic and can vary in prevalence across different genetic lineages. Pfam annotation of all antitoxins groups has been provided in File S5.

Out of the four highly prevalent (≥80%) toxin groups predicted in our study, the CptA_toxin (2H) (cytoskeletal polymerization inhibiting) group was observed to be associated with three different partners [CptA_toxin (2H)/UNKW (3P), Sdh5 (2P)/CptA_toxin (2H)/UNKW (3P), and Sdh5 (2P)/CptA_toxin (2H)/YgfX (264P)]. As apparent from the name, CptA exerts an inhibitory effect on the polymerization of cytoskeletal proteins MreB and FtsZ (22). As a result, they are implicated in growth inhibition and morphological changes in bacteria. Of the three genetic orientations, Sdh5 (2P)/CptA_toxin (2H)/UNKW (3P) was more common; the other two orientations were rarely prevalent (2%) and found in ST405 only.

Approximately 62.72% of the hits were found to be associated with single partners while approximately 37.28% were observed to be associated with more than or equal to two partners (File S5). Additionally, our analysis revealed that certain antitoxin partners within TA operons display ST-specific or phylogroup-specific prevalence patterns. For example, YafO_toxin (79P)/Fic (59H) (Fig. 6C) operon was exclusively present in ST117. A detailed view of TA structures and lineage preference of associated partners is given in Fig. S3.

### Comparison of antitoxin hits with TADB and TASmania (novel vs. known antitoxins)

A detailed view of known and novel statuses has been provided in Table 1. Of the 290 antitoxins predicted, 181 (62.41%) antitoxins were observed to be novel, while the rest of them were observed to be known antitoxins. Antitoxins were functionally annotated using interpro-scan with reference to Pfam and NCBIfam databases (30). As compared with Pfam, only four additional hits were observed with NCBIfam, i.e., UNKW (11P) as cell division protein DrpB, UNKW (74P) and UNKW (159P) as the host cell inhibitor ICD domain, and UNKW (213P) as adhesin of bacterial autotransporter systems. Among 181 newly identified antitoxins, 54 were assigned potential functions. Among these Ub-RnfH (1P) and DMT_YdcZ (5P) were widespread (>95%) across all the STs. Similarly, CcdB (58P) recognized as one of the novel antitoxins was found in 100% of the strains belonging to ST117 and ST38. Other distinctive novel markers such as YafO (76P) and YafO (77P) were observed exclusively among ST69.

**TABLE 1:** A list of toxin Pfam domains with number of associated hit clusters, partner clusters, and proportion of known antitoxins

S. no.	Pfam domain	Hit clusters	Partner clusters	Known (proportion)
1	PIN	4	7	0.57
2	AntA	4	10	0
3	HicA_toxin	9	10	0.80
4	Zeta_toxin	4	4	0.25
5	ParE_toxin	21	36	0.56
6	PemK_toxin	8	17	0.29
7	RES	2	2	0.0
8	YafO_toxin	6	13	0.08
9	DUF955	4	5	0.40
10	HigB-like_toxin	7	11	0.55
11	Bro-N	7	11	0.18
12	YdaT_toxin	7	37	0.35
13	HD	3	5	0.20
14	ANT	6	10	0
15	HigB_toxin	3	4	0.75
16	RelE	6	11	0.73
17	GnsAB_toxin	1	1	1
18	CbtA_toxin	6	17	0.24
19	GNAT_acetyltran	2	5	0
20	Gp49	11	14	0.57
21	CcdB	6	10	0.6
22	AbiEii	2	3	0
23	Toxin_YhaV	1	7	0.14
24	YoeB_toxin	1	1	1
25	YjhX_toxin	1	2	0
26	Fic	8	12	0.58
27	DUF4258	1	1	0
28	Polyketide_cyc2	2	2	0
29	NTP_transf_2	2	2	0
30	HipA_C/Couple_hipA	1	6	0.17
31	Cpta_toxin	1	3	0.33
32	MqsR_toxin	1	1	1
33	HipA_C	4	5	0.6
34	YafQ_toxin	2	3	0.33
35	HD_3	2	2	0

### Orphan antitoxins and toxins

Our analysis was extended beyond the prediction of the entire TA module and encompassed the investigation of orphan toxins and antitoxins (toxin or antitoxin lacking its corresponding antitoxin or toxin counterpart) across the entire data set of 950 genomes. For toxin sequences, the minimum amino acid length was set to 30 and the maximum amino acid length was set to 200. These criteria cover approximately 90% of the sequences from TADB. Likewise, for antitoxin sequences, our selected criteria were a minimum amino acid length of 50 and a maximum amino acid length of 200, covering approximately 95% of antitoxin sequences from TADB. By targeting the range of lengths that covers approximately 90% toxin sequences and 95% antitoxin sequences, we ensure that our analysis represents majority of data (Fig. S5). Orphan antitoxins were found to be highly abundant across all STs. Among the 290 antitoxin groups that were predicted, we identified 176 groups as unpaired, with a total occurrence of 73,201. In certain cases, it was observed that when specific antitoxin partners were not widely detected, there was a contrasting prevalence observed among orphan antitoxins (Fig. S6). For instance, for the UNKW (6P) antitoxin, only 12% of the isolates were found to be paired, while 88% of the isolates were observed to have UNKW (6P) as an orphan antitoxin (Fig. S6). Similarly, orphan toxins were also found to be highly abundant across all sequence types (Fig. S7). Among the 169 toxin groups predicted, 113 groups were identified as unpaired, amounting to a total of 40,506. In contrast to orphan antitoxins, orphan toxins tended to be ST specific/phylogroup specific, indicating a more targeted distribution.

### Co-occurrence patterns with other genetic coordinates

**Fig 7:**
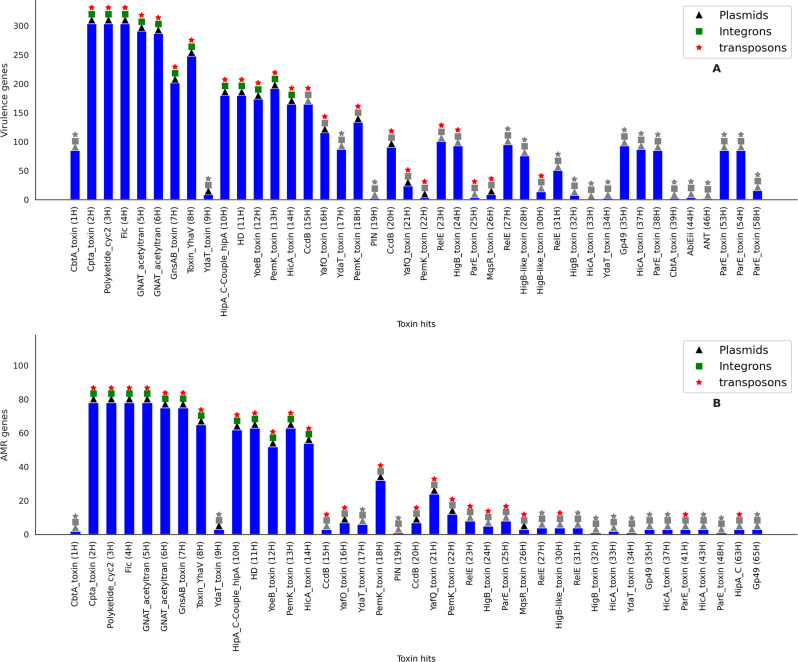
Bar plots depicting the association of toxin groups with (A) virulence genes and (B) antimicrobial resistance encoding genes. Symbols placed over the bars—triangle (black), square (green) and asterisk (red)—indicate the respective associations of plasmids, integrons and transposons with toxin groups. The gray colored symbols, however, signify absence of a significant association.

TA systems have been frequently linked to stress responses in *E. coli*. Hence, we attempted to investigate the co-occurrence of toxin genes with AMR genes, virulence genes, and other genetic markers prone to horizontal gene transfer (HGT). We performed Fisher’s exact test to find out whether there was a significant co-occurrence of toxin genes with the above-mentioned genetic coordinates. Our analysis found that 42 toxin hits had significant co-occurrence patterns with virulence genes (Fig. 7A). However, not much can be deduced for toxin groups that are highly prevalent. Nevertheless, hits restricted to a single ST, such as ParE-toxin (58H) in ST11, showed significant co-occurrence with a distinct set of virulence genes encoding a type III secretion system, a Shiga-like toxin (*toxB*), and tir-cytoskeleton coupling protein-encoding gene (*tccP*). As previously reported, this particular set of genes is exclusive to ST11 (16).

Additionally, we observed that 39 toxin hits showed significant co-occurrence with AMR-encoding genes (Fig. 7B). Among these 39 toxin hits, 19 were associated with plasmids, 12 with integrons, and 25 with transposons, in the genomes of *E. coli* bacteria.

## DISCUSSION

*E. coli* is renowned for its remarkable genomic plasticity and adaptability, enabling it to thrive in ecologically diverse environments. One intriguing aspect of *E. coli’*s genomic landscape is the abundance of TA systems. These TA structures, found in *E. coli* and other clinically relevant pathogens, have been suggested to be implicated in a wide range of biological processes encompassing biofilm formation, stress responses, AMR, and persister formation (14). In the present study, we attempt to understand the prevalence, diversity, and evolutionary patterns of TA systems across 19 different clonal lineages of *E. coli*. Genomes used in this study were ranked and classified (16) into 19 different STs based on machine learning algorithms. Genomic fine-tuning and niche-specific adaptation events may lead to variations even among the closely related members of the same species (31). The current study, encompassing large-scale WGS-based computational genomics unveiled considerable diversity in the prevalence of TA systems across different lineages as well as their evolutionary relatedness as depicted in PCoA (Fig. 2). Specifically, the comparative genomics analyses at the ST level showed that ST12 exhibits a reduced toxin repertoire compared with other STs (Fig. 1D). Moreover, at the phylogroup level, phylogroup B2 (ST12, ST127, ST73, ST95, and ST131) and phylogroup C (ST410) displayed a diminished pool of toxins (Fig. 3). Notably, one strain from ST12 with draft genome status was completely devoid of toxin genes, suggesting a possibly unique selection pressure leading to a complete loss of toxin genes in that particular strain. Our findings concerning the reduced toxin repertoire of phylogroup B2 are consistent with earlier studies (2, 17, 20). However, our observations based on eight different phylogroups of *E. coli* and the constituent lineages appear to be uniquely decoding the phenomenon of reduced toxin repertoires indicative of differential selection pressures. Recent studies indicate that the epidemiological success of ST410 can be attributed to evolutionary events similar to that of ST131 (3). Therefore, our observations further support the gene loss hypothesis in the case of ST131 and ST410 to maximize fitness and drive the evolution of specialized lineages (2, 32).

The diminished TA repertoire of phylogroup B2 and the higher repertoire of TA systems in phylogroup B1 and phylogroup A have been previously linked to their lifestyle and host preferences (17). Generalist lineages (phylogroup B1 and phylogroup A) can inhabit a wide range of ecological niches, making them more vulnerable to stressful situations. As a result, context-dependent activation of TA systems may help in their survival and adaptation to diverse conditions. Specialist lineages, such as those belonging to phylogroup B2, primarily known as ExPECs (extraintestinal pathogenic *E. coli*), do not face significant environmental fluctuations and thus do not require specialized mechanisms to cope with stressful situations. As a result, genetic elements that may be detrimental to pathogenic lifestyles in specialist lineages may gradually disappear over time. Nevertheless, genome reduction cannot be solely attributed to the epidemiological success of high-risk clones such as ST131. Instead, a combination of factors could drive the evolution and global expansion of such lineages (2, 16, 33).

Overall, four toxin groups [CptA_toxin (2H), Polyketide_cyc2 (3H), Fic (4H), and GNAT_acetyltransferase (5H)] were observed to be highly prevalent (≥80%) across all the studied STs (Fig. 3). Among these toxin groups, CptA_toxin (2H), Fic (4H), and GNAT_acetyltransferase (5H) were associated with multiple partners in different genetic orientations. However, a preference for a particular partner/genetic orientation was observed in the case of CptA_toxin (2H) and Fic (4H). In contrast, in the case of GNAT_acetyltransferase (5H), ST-specific prevalence was observed for UNKW (51P)/CptA_toxin (5H)/DMT_YdcZ (5P) (ST117, ST69, and ST405), while others were prevalent for GNAT_acetyltransferase (5H)/DMT_YdcZ (5P). Although the above four toxin groups were highly prevalent across STs, some were restricted to specific phylogenetic backgrounds. Similar to those of toxin groups, antitoxins including Ub-RnfH (1P), Sdh5 (2P), UNKW (3P), YhfG (4P) and DMT_YdcZ (5P) were highly prevalent across all the STs (Fig. S2). Among these highly prevalent antitoxins, RnfH (1P), UNKW (3P), and DMT_YdcZ (5P) were identified as novel and previously uncharacterized, deserving further investigation. Moreover, among the novel antitoxins, only 54 (29.83 %) of them could be assigned with potential functions. This observation highlights the need of further functional studies uncovering the mechanisms and biological significance of these novel antitoxins.

The conservation of TA systems across diverse genetic backgrounds in *E. coli* may be attributed to the vertical transmission of these elements (12). Similarly, in the case of the *K. pneumoniae* species complex, there is an evidence in favor of a similar conservation pattern concerning TA repertoire, where toxin groups of the same species exhibit higher nucleotide identity than members of different species (18). The conserved prevalence patterns and copy numbers of TA systems in *P. syringae* have been suggested to play an important role in *P. syringae* ecology (19). These findings suggest that in *E. coli*, similar to that of *K. pneumoniae*, the conserved abundance of toxin repertoire can be attributed to vertical transmission and selective pressures that maintain TA systems in specific genetic backgrounds. In addition to the conserved patterns, certain toxin groups were found in different phylogenetic backgrounds with varying degrees of prevalence. This variation in prevalence may be attributed to multiple factors, including their mobile nature or susceptibility to horizontal gene transfer, as well as host and environmental factors.

Gene dosage increases due to genome duplication and amplification, with possible implications in bacterial adaptation in diverse environmental contexts (34). Moreover, gene amplification can also impact human disease by modifying antibiotic resistance and virulence (35). In our study, the tendency of having a higher copy number of toxin groups in certain STs suggests their potential role in adaptation and selection pressure favoring their retention (Fig. 4). Further, experimental investigations are crucial to establish the mechanisms driving copy number variation across different phylogenetic backgrounds and their potential functional implications.

In our data, we observed an abundance of orphan toxins/antitoxins functionally not linked to their original partners (Fig. S6, Fig. S7). There have been multiple lines of evidences entailing orphan antitoxins/toxins in literature (36–38). *Neisseria gonorrhoeae*, the causative agent of gonorrhea, carries a split TA system composed of an orphan toxin VapD encoded on pConj and antitoxin VapX encoded by other mobile genetic elements. pConj conserved across *N. gonorrhoeae* strains harbors genes encoding resistance against beta-lactams and tetracyclines, and it is possible to use interventions targeting the functioning of this split TA system to overcome the harm posed by this plasmid in gonococcus (37). Orphan toxins, such as OrtT in *E. coli*, have been implicated in their ability to alleviate the stress associated with amino acid and DNA synthesis.

Similarly, orphan antitoxins have the potential to perform numerous functions in bacterial cells. They may function as anti-addiction molecules (protection of bacterial cell from PSK by plasmid-encoded counterparts), transcriptional regulators of other TA systems, and specific genes in the bacterial genomes (39). In the example case of the UNKW (6P) antitoxin group, only 12% of the isolates were found to be paired, while 88% of the isolates were observed to have UNKW (6P) as an orphan antitoxin (Fig. S6). This observation suggests a trans-acting role for antitoxins. However, it is important to note that these assumptions should be validated and investigated through experimental studies to understand the precise role of orphan antitoxins in bacterial physiology. Since the prediction of TA systems in this study was based on some draft genome sequences and pre-defined structural parameters, there could be a possibility that orphan toxins/antitoxins predicted in our study were originally related to their partners, but this possibility was ruled out due to the parameters (18) that we utilized in this study. Additionally, the abundance of orphan antitoxins in members of phylogroup B2, such as ST131 isolates (100% complete genomes), could be potentially attributed to gene loss that perhaps made them tidier and clonal. Strains of the ST131 group are known super-bugs posing very high risk of clonal transmission among different settings.

Furthermore, it is important to note that the copy number and orphan toxins/antitoxins can be influenced by the quality and finish of the genomes. Hence, the probability of the copy number and existence and functions of orphan toxins/antitoxins would need further genomic and experimental validation and refinement.

In conclusion, our study entails a rigorous effort to conduct a large-scale, multi-parametric, comparative genomic analysis of TA systems across diverse genetic lineages of *E. coli*. Based on our findings, we hypothesize that *E. coli* has undergone numerous evolutionary events leading to an abundance of TA structures in its genomes. These TA systems are likely to have a significant role in the evolutionary trajectory of high-risk clones, influencing their survival, adaptation, persistence, and resistance in different contexts (host and other environmental factors). Our study also provided working evidence for the presence of genetic coordinates that have a significant association with the marker toxin groups. Going forward, these observations would be complemented by molecular studies to understand the activation, regulation, and functional dynamics of TA systems under different environmental conditions entailing pathogenic bacteria, specifically, *E. coli*.

## MATERIALS AND METHODS

### Selection and curation of genomic data

*Escherichia* resource data of 5,653 strong *E. coli* genomes, as described by our group earlier (16), was harnessed for this study, whereby, 50 representative genomes from each of the 19 STs were recruited. Random sampling was used to choose genomes for analysis in order to mitigate potential sampling biases. However, inherent biases from geographical location, isolation source, host, and other factors may still exist in our data set. The selection strategy was planned to prioritize complete genomes over draft ones. Genomes were categorized into draft and complete based on NCBI metadata. Assemblies labeled as “complete” were considered fully sequence genomes, while assemblies labeled as “scaffolds” or “contigs” were considered as drafts. If the count of the complete genome per ST was exceeding 50, a random selection of the 50 genomes was made, and if this count was less than 50, good-quality draft genomes were pulled in to make a uniform count of 50 genomes per ST, totaling to 950 genomes. The metadata of the selected genomes has been provided in File S1.

### Prediction of toxin-antitoxin systems and statistical analysis

SLING version 2.0.1 (21), an open-source Python-based command-line tool, was used to predict toxins and their cognate antitoxins using an in-built toxin domain database. TA systems were predicted through a four-step process, i.e., genome preparation, scanning of input genomes to identify HMM hits (toxins), filtering to identify cognate partners (antitoxins), and grouping of hits, partners, and discarded hits. In the preparatory step, complete or draft genomes and respective annotation files of the respective assemblies in GFF format were used as input. Followed by toxin (hit) and antitoxin (partner) search, filtering and grouping of hits were carried out. Filtering was done based on certain thresholds, such as a minimum hit length of 30 aa, maximum hit length of 200 aa, minimum partner length of 50 aa, maximum hit length of 150 aa, and a maximum overlap of 20 bp. Potential antitoxins were annotated by interproscan using Pfam database (30). Exploratory data analysis of predicted toxins, antitoxins, and toxin-antitoxin pairs was performed to understand their distribution across STs using in-house Python scripts.

The percent prevalence of toxins, antitoxins, and complete TA pairs was calculated using the formula number of toxins or antitoxins or T-A pairs present in genomes of a particular ST/total number of genomes in a particular ST (*n* = 50). Toxins/antitoxins/TA pairs with at least 20% presence in any STs under investigation were plotted using matplotlib and seaborn libraries of Python.

### Principal coordinate analysis

PCoA based on the Jaccard distance matrix of the binary matrix representing toxin repertoires was carried out to investigate the ST-specific clustering pattern (genetic diversity) of strains. To assess the genetic relatedness among strains and dissimilarities among STs, PERMANOVA was performed.

### Genetic organization of TA operons

SLING-generated group files of antitoxin homologs were used to mine the location of associated antitoxin using in-house-written Python scripts.

### Prediction of orphan antitoxins and orphan toxins

Orphan antitoxins and toxins were predicted using a similar approach based on the methodology described by Horesh et al. (18).

Briefly, for the prediction of orphan antitoxins, antitoxin sequences were grouped using CD-HIT Version 4.8.1. with default parameters of 90% identity and 5-kmer size (40). Subsequently, a single FASTA file with grouped sequences was used to create an antitoxin nucleotide database using BLAST 2.9.0+ (41). Next, the entire data set comprising 950 high-quality assemblies was aligned against the antitoxin database using BLASTn with default parameters. The criteria for considering a coding sequence (CDS) as an orphan antitoxin were as follows: a minimal length of 50 aa, a maximum length of 150 aa with 75% identity, and an alignment length of at least 50 aa.

Similarly, for the prediction of orphan toxins, the toxin sequences were grouped using CD-HIT version 4.8.1 with the same default parameters used for antitoxin clustering (90% identity and 5-kmer size) (40). The clustered sequences were then used to create a toxin nucleotide database using BLAST 2.9.0+ (41). The data set comprising 950 high-quality assemblies was aligned against the toxin database using default parameters. Criteria similar to that of the original prediction were used to declare a CDS sequence as an “orphan toxin.” The criteria for considering a CDS as an orphan toxin were as follows: a minimal length of 30 aa, a maximum length of 200 aa with 75% identity, and an alignment length of at least 30 aa.

### Unraveling functional variants based on the experimental database (novel vs. known antitoxins)

The amino acid sequences of both type II and type IV, which were computationally predicted and experimentally validated, were obtained from TADB 2.0 (42). All the validated sequences were clustered using CD-HIT Version 4.8.1 with default parameters of 90% identity and 5-kmer size (40). In the following step, the above-clustered sequences were used to create an antitoxin protein database using BLAST 2.9.0+ (41). Subsequently, BLASTx was carried out to align the antitoxins predicted with TADB. A predicted antitoxin was considered “known” if its identity was 85% or higher, coverage was 85% or higher, and the e-value was 0.01 or lower. Further, antitoxins were also screened against TASmania database (43). Briefly, antitoxin HMM profiles were downloaded from TASmania web-server, and then, hmmsearch was used to predict antitoxins with e-value 0.01 or lower. If appropriate match was not detected in either of the aforementioned databases, then antitoxin was categorized as “novel;” otherwise, it was classified as “known.”

### Co-occurrence patterns with other genomic coordinates

Co-occurrence patterns of toxins with other genomics coordinates, including antimicrobial resistance, virulence genes, plasmids, integrons, and transposons, were studied using Fisher’s exact test (FDR corrected, alpha = 0.01). AMR encoding genes and virulence genes were predicted using ABRicate (https://github.com/tseemann/abricate) with minimum identity and coverage of 80% against CARD (44) and VFDB (45) databases, respectively. PlasmidFinder (46) was used to predict plasmids with default parameters, while BacAnt (47) was used to predict integrons and transposable elements in the genomes of the genus *Escherichia*.

## SUPPLEMENTAL MATERIAL

File S1 mbio.03023-23-s0001.xlsx NCBI metadata of the genomes under analysis.

File S2 mbio.03023-23-s0002.pdf Abbreviations.

File S3 mbio.03023-23-s0003.xlsx Toxin presence/absence, toxin count per strain, percent prevalence, and statistics.

File S4 mbio.03023-23-s0004.xlsx Copy number variation in toxin hits.

File S5 mbio.03023-23-s0005.xlsx Antitoxin count associated with different toxin hits and Pfam annotation.

Supplemental Figures mbio.03023-23-s0006.pdf Figures S1 to S7.

ASM does not own the copyrights to Supplemental Material that may be linked to, or accessed through, an article. The authors have granted ASM a non-exclusive, world-wide license to publish the Supplemental Material files. Please contact the corresponding author directly for reuse.
