# Supplementary material for: Unraveling the evolutionary dynamics of toxin-antitoxin systems in diverse genetic lineages of Escherichia coli including the high-risk clonal complexes
Source: mBio. 2023 Dec 20;15(1):e03023-23. doi: 10.1128/mbio.03023-23 (PMC10790755; doi:10.1128/mbio.03023-23)
Supplement: File S2 — Abbreviations. [file mbio.03023-23-s0002.pdf]

Supplementary File 1

Table: List of key terms (abbreviations) used in the manuscript

S. No	Term	Description
1	H	Hit (toxin group)
2	P	Partner (antitoxin group)
3	T_AT	Toxin-Antitoxin
4	AT_T	Antitoxin-Toxin
5	AT-T-AT	Antitoxin-Toxin-Antitoxin
6	UNKW	Unknown
