# Supplementary material for: Unraveling the evolutionary dynamics of toxin-antitoxin systems in diverse genetic lineages of Escherichia coli including the high-risk clonal complexes
Source: mBio. 2023 Dec 20;15(1):e03023-23. doi: 10.1128/mbio.03023-23 (PMC10790755; doi:10.1128/mbio.03023-23)

Unraveling the Evolutionary Dynamics of Toxin-Antitoxin Systems in Diverse Genetic Lineages of *Escherichia coli* including the High-risk Clonal Complexes

Anuradha Singh, Aditya Kumar Lankapalli, Suresh Kumar Mendem, Torsten Semmler, Niyaz Ahmed

Supplementary Figures

Detailed captions/legends

Fig. S1: Scree plot depicting the explained variance of top 10 principle coordinates.

Fig. S2: Heatmap illustrating the percentage prevalence of antitoxin groups across 19 STs of *E. coli*. The x-axis depicts ST lineage, while the y-axis depicts antitoxin groups identified. The color bar on the right side depicts % presence.

Fig. S3: Heatmap illustrating the percentage prevalence of TA pair across 19 STs of *E. coli.* The x-axis depicts ST lineage, while the y-axis depicts TA pair identified. The color bar on the right side depicts % presence.

Fig. S4: Toxin-antitoxin network of *E. coli* isolates with red colored nodes depicting toxin hits and grey colored nodes depicting antitoxin partners, while edges represent association between TA pairs.

Fig. S 5: Distribution plots for the range of amino acid lengths selected for toxin/antitoxin prediction.

Fig. S6: Heatmap illustrating the percentage prevalence of orphan antitoxins across 19 STs of *E. coli*. The x-axis depicts ST lineage, while the y-axis depicts orphan antitoxin identified. The color bar on the right side depicts % presence.

Fig. S7. Heatmap illustrating the percentage prevalence of orphan toxins across 19 STs of *E. coli*. The x-axis depicts ST lineage, while the y-axis depicts orphan toxin identified. The color bar on the right side depicts % presence.

Fig. S1: Scree plot

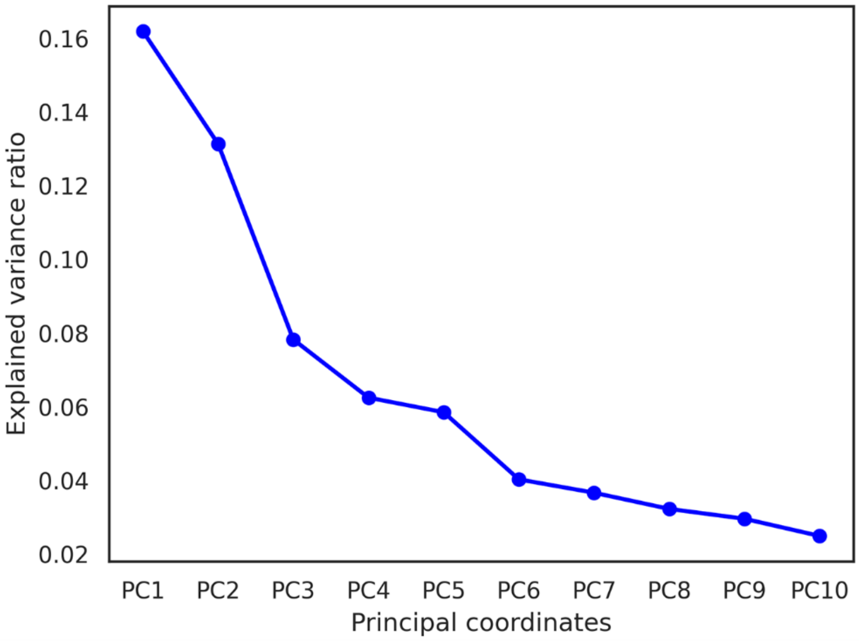

Fig. S2: Heatmap depicting prevalence of antitoxin groups

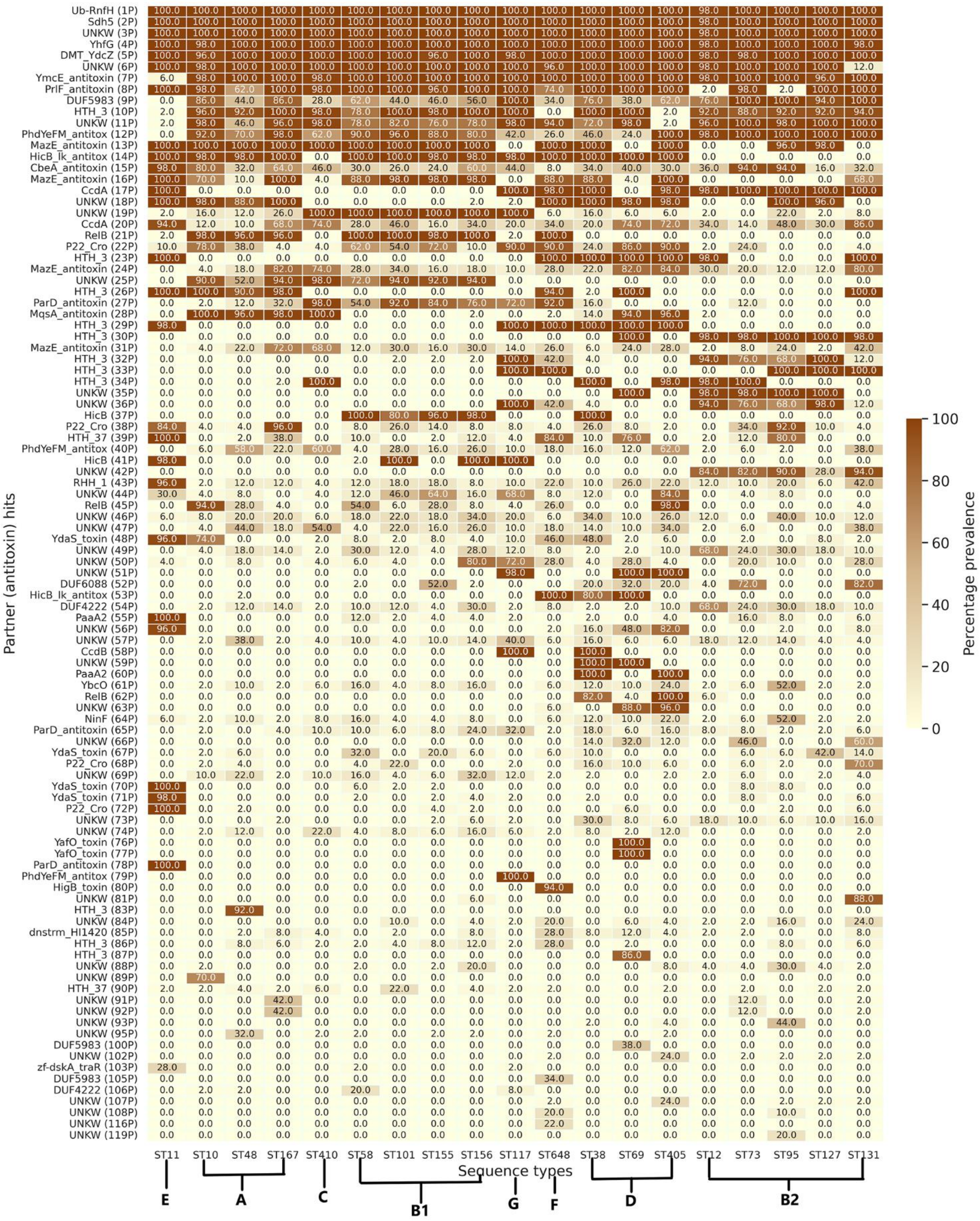

Fig. S3: Heatmap depicting prevalence of complete TA operons

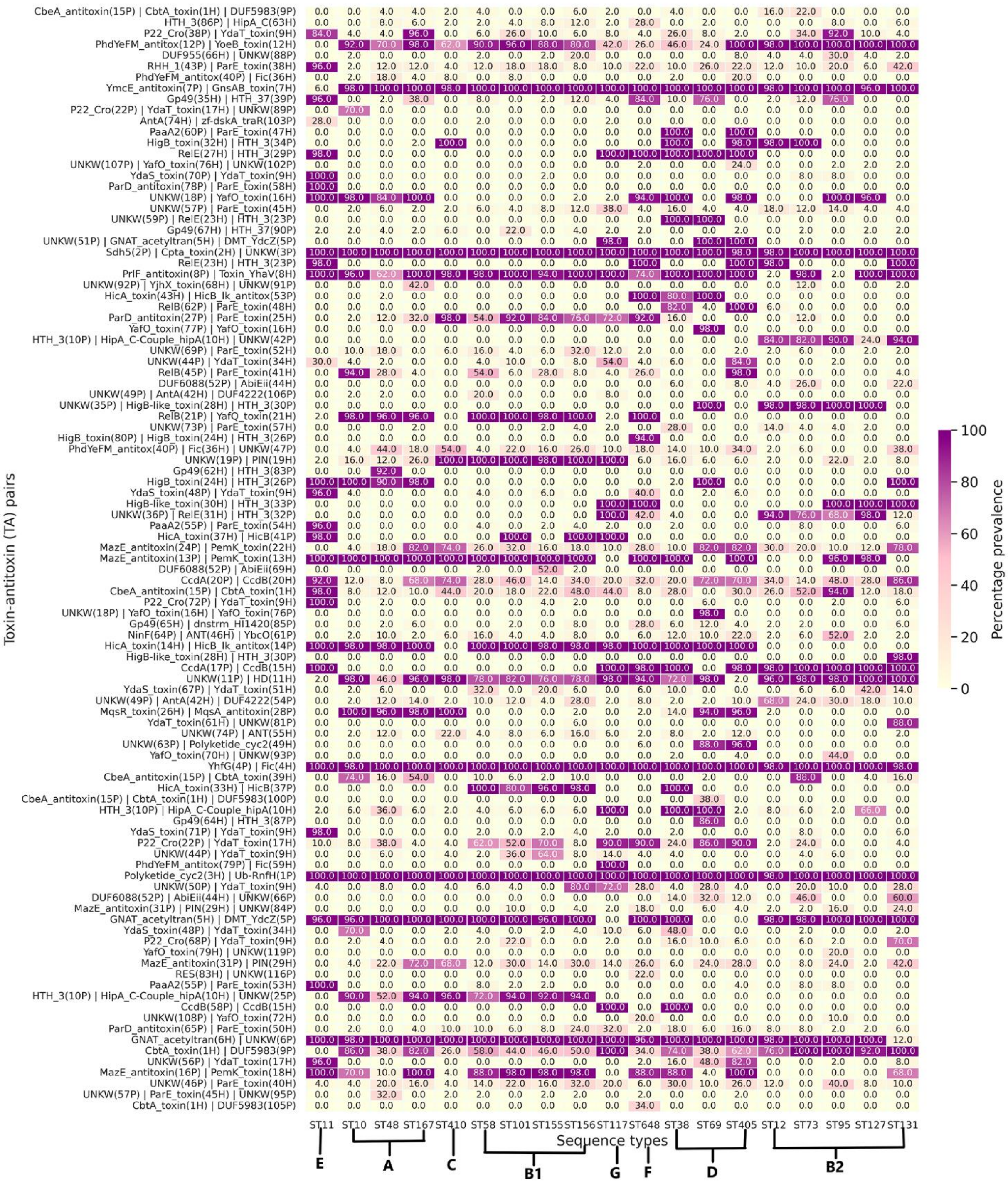

Fig. S4: Toxin-antitoxin network graph

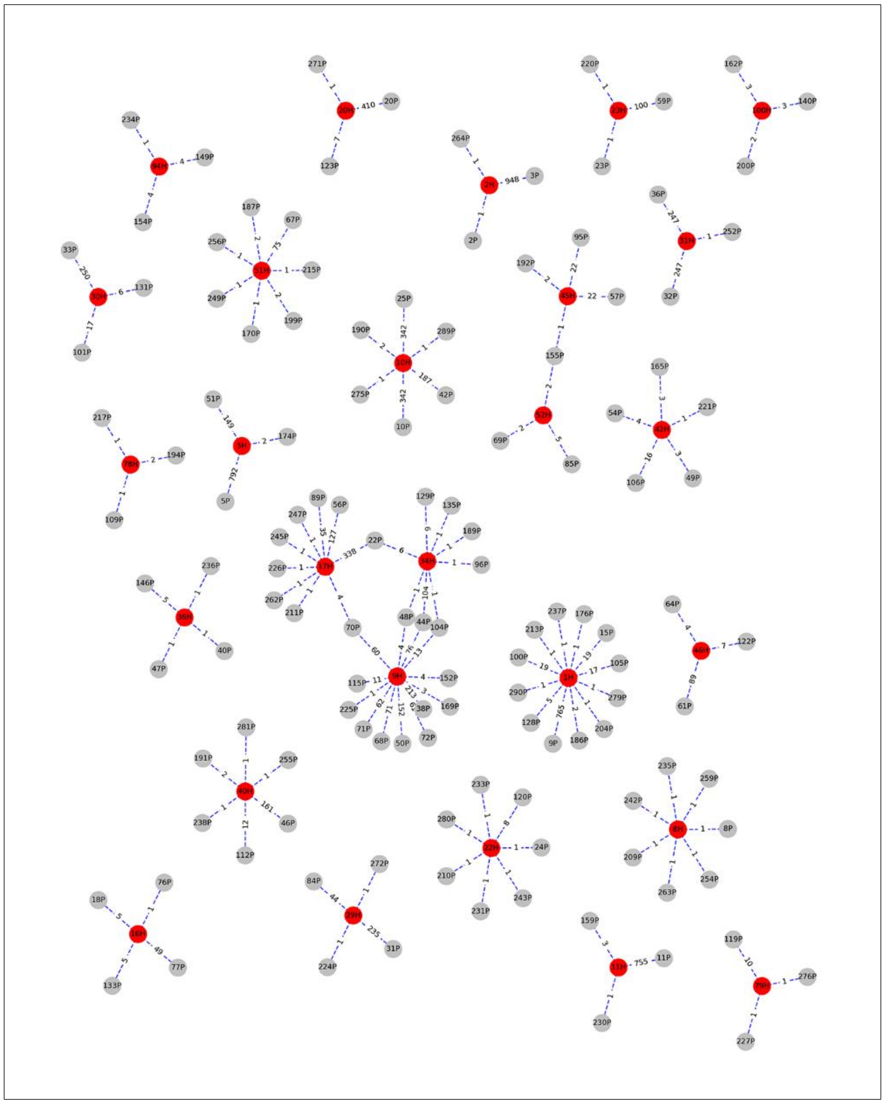

Fig. S5: Distribution plots of amino acid length selected for TA systems prediction

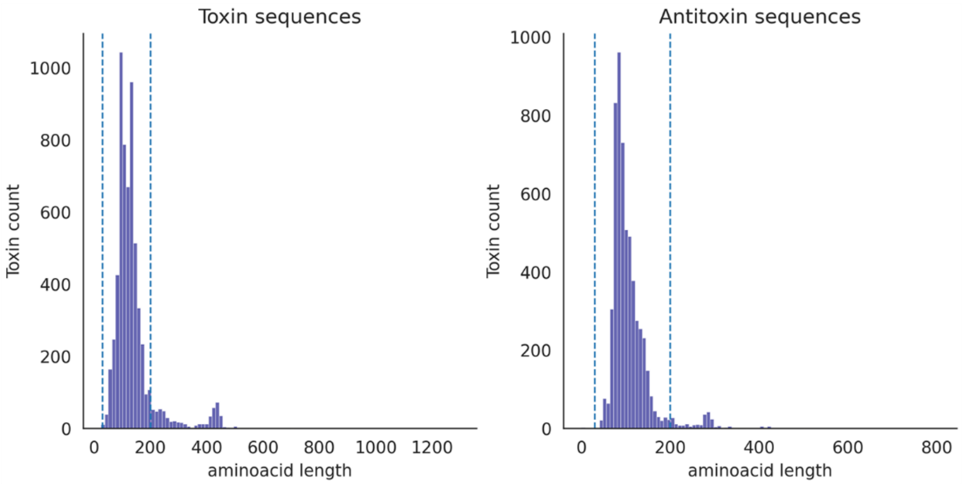

Fig. S6: Heatmap depicting prevalence of orphan antitoxins

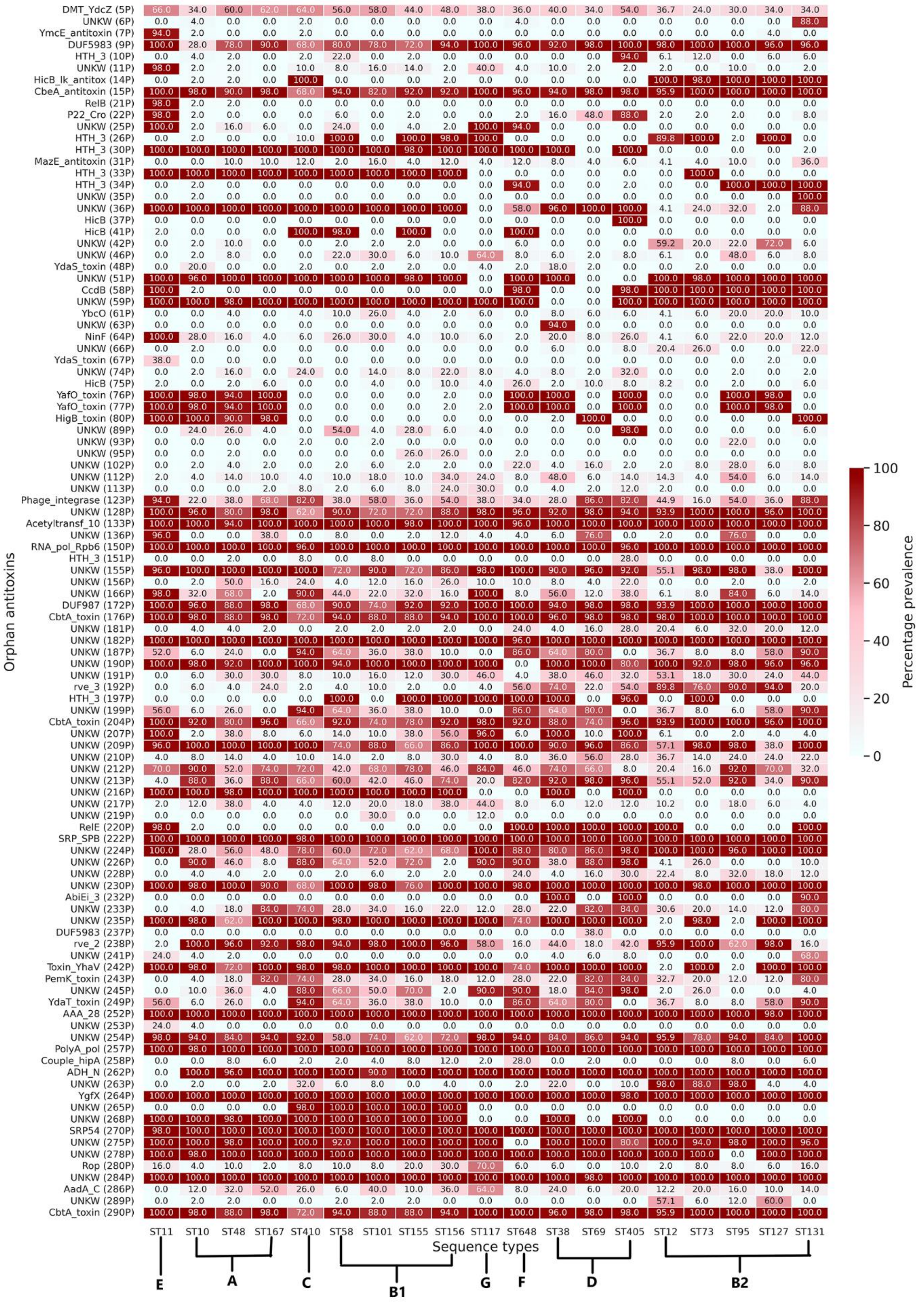

Fig. S7: Heatmap depicting prevalence of orphan toxins

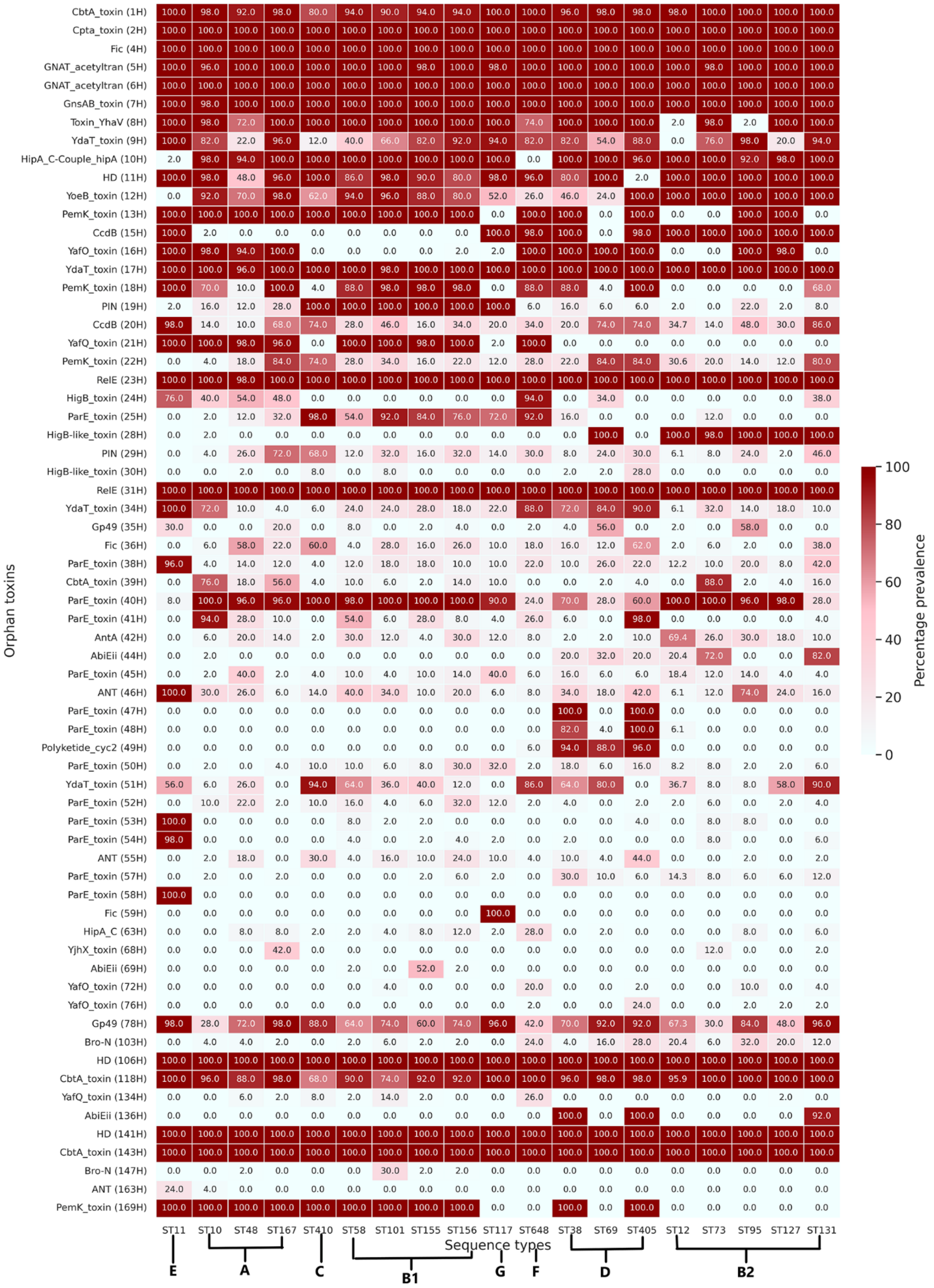

Supplement: Supplemental Figures — Figures S1 to S7. [file mbio.03023-23-s0006.pdf]
